# A dataset about anthropometric measurements of the Pakistani children and adolescents using a cross-sectional multi-ethnic anthropometric survey

**DOI:** 10.1016/j.dib.2020.106642

**Published:** 2020-12-11

**Authors:** Muhammad Asif, Muhammad Aslam, Muhammad Qasim, Saima Altaf, Amir Ismail, Hamza Ali

**Affiliations:** a Department of Statistics, Govt. Degree College Qadir Pur Raan, Multan Pakistan; b Department of Statistics, Bahauddin Zakariya University, Multan Pakistan; c Department of Economics, Finance and Statistics, Jönköping University, Jönköping, Sweden; d Institute of Food Science and Nutrition, Faculty of Agricultural Sciences, Bahauddin Zakariya University, Multan Pakistan; e Department of Zoology, Govt. Degree College Qadir Pur Raan, Multan Pakistan

**Keywords:** Anthropometric measurements, Children and adolescents, Nutritional status, Obesity, Pakistan, Self-administered questionnaire

## Abstract

Evaluation of nutritional status is necessary during childhood and the juvenile years when the level of hydration and the adipose tissues experience significant changes. Anthropometric measurements and their derived indices are valid proxies to predict body fat, obesity (general or central) and their associated cardiovascular risks. The dataset under consideration also provides the socio-demographic related information and anthropometric measurement values related to height, weight, body mass index (BMI), waist circumference (WC), hip circumference (HpC), waist-to-hip ratio (WHpR), waist-to-height ratio (WHtR), mid-upper arm circumference (MUAC), neck circumference (NC), and wrist circumference (WrC). Standard procedure was adopted for quantifying the body measurements. The data were consisting of 10,782 children and adolescents aged 2–19 years, belonging four major cities of Pakistan viz. Multan, Lahore, Rawalpindi and Islamabad. This dataset is beneficial to develop anthropometric growth charts which will provide the essential knowledge of growth and nutritional disorders (e.g., stunted, overweight and obesity) of Pakistani children and adolescents. The dataset can also be used by researchers to calculate body surface area (BSA), body frame size (BFS), body shape index (BSI), and tri-ponderal mass index (TMI) of children and adolescents that are also some other reliable indicators of obesity and insulin resistance as well as cardiometabolic risk in children and adults.

## Specifications Table

**Table:** 

Subject	Children and Adolescents Health
Specific subject area	Child Health and Nutrition
Type of data	Primary data, Tables
How data were acquired	The data were collected using a self-administered questionnaire and converted into the .xlsx format. A copy of the self-administered questionnaire is incorporated in the supplementary file. Anthropometric measurements of every individual were collected using standard equipment i.e., height was taken on stadiometer (Seca: SCA217), weight was measured using a weighing machine (Westpoint WF 7009).
Data format	The data were in raw format and it was converted into an MS-Excel (xlsx) format.
Parameters for data collection	Socio-demographic related-information i.e., age (years), gender status (boys/girls), residential city (Multan/ Lahore/ Rawalpindi or Islamabad) and anthropometric measurements i.e., height (cm), weight (kg.), WC (cm), HpC (cm), MUAC (cm), NC (cm) and WrC (cm) of children and adoelscents were collected using standard techniques.
Description of data collection	The cross-sectional survey type data were collected from 10,782 children and adolescents, aged 2 to 19 years, who belonged to four major cities of Pakistan including Lahore (a capital city of Punjab), Multan (located in the south of Punjab and is also identified at the central point of Pakistan's map), Rawalpindi, and Islamabad is the capital city of Pakistan. The studied children and adolescents aged 4 to 19 years were sampled from randomly selected public and private schools. The complete list of schools (primary, secondary and higher secondary) of the respective cities was taken from Punjab Department of Education (schools). The rest of the data for the children of age-group 2 to 4 years were collected from different public places (i.e., parks, shopping malls, markets, etc.).
Data source location	Region: Asia Country: Pakistan
Data accessibility	The dataset can be accessed through the following link: https://data.mendeley.com/datasets/sxgymx5xjm/1

## Value of the Data

• To the best of our knowledge, this is the first dataset about the various number of anthropometric measurements for the Pakistani children and adolescents. We provide the socio-demographic related information and anthropometric measurement values related to height, weight, body mass index (BMI), waist circumference (WC), hip circumference (HpC), waist-to-hip ratio (WHpR), waist-to-height ratio (WHtR), mid-upper arm circumference (MUAC), neck circumference (NC), and wrist circumference (WrC) of all subjects under dataset.
• All pediatricians, epidemiological researchers, nutrition experts, and policymakers can get benefit from our anthropometric measurements dataset to develop growth charts which would provide the essential knowledge of growth and nutritional disorders (e.g., stunted, overweight and obesity) of the Pakistani children and adolescents.
• Analysis of dataset will be valuable for the researchers who want to compare age- and gender-specific prevalence of thinness, overweight, obesity, central obesity and body fat of the Pakistani children and adolescents with other developing and developed countries datasets.
• Different health professionals also recommended different body circumferences (i.e., MUAC, WC, HpC NC and WrC) and their derived indices (waist-to-hip ratio (WHpR), waist-to-height ratio (WHtR); arm-to-height ratio (AHtR); height-to-wrist ratio (HWrR) etc.) to use as indicators for clinical evaluation of nutritional and cardiometabolic disorders. The present dataset is of value to those researchers who want to conduct a research to assess nutritional, cardiometabolic and cardiovascular disorders. All the institutions pertinent to child health and pediatric endocrinology & metabolism can benefit from these data.
• This dataset is beneficial for the local researchers to construct the ethnic-specific anthropometric growth charts that would be used for growth monitoring and nutritional status assessment of children and adolescents. The data can also support national and international health agencies around the world to make the comparison of anthropometric characteristics related to the growth and development among children and adolescents.
• Our data also provide a wealth of information about body dimensions, which are useful for the development of sizing systems for clothing, manufacturing of many medical equipments, and some special furniture etc.

## 1 Data Description

The activity of data collection was completed during March to June 2016 by three well-trained data collection teams, supervised by the principal investigator. A self-administered questionnaire was designed and divided into two parts. The first part includes socio-demographic information, for instance, gender status (boys/girls); residence city (Multan/Lahore/ Rawalpindi or Islamabad) and age (rounded to next year) of children. Age was confirmed from the school register with the assistance of class teacher and children whose measurements were taken from public places, their ages were confirmed from their parents/guardians at the time of interview. Anthropometric measurements of each individual were taken [3,4] and recorded in the second part of the questionnaire. The variables height (cm) without shoes was taken by a stadiometer (Seca: SCA217) and weight (kg) in light cloths was measured using a weighing machine (Westpoint WF 7009). While body circumferences (to the nearest 0.1 cm) were measured using a non-stretchable plastic tape without squeezing the skin. During these measurements, the subject was in the comfortable standing position and they were asked to look straight ahead with shoulders in the normal position. BMI of an individual was obtained by weight in kilogram divided to the square of his height in meters (BMI=Kg/m^2^). The WHpR and WHtR were obtained by dividing WC to HC and WC divided to height, respectively. All the normal children and adolescents 2–19-year-old, who were not taking any medication,not having any physical disability and not affected by any diseases were included in the dataset. All children who did not meet these criteria were excluded.

**Fig. 1:**
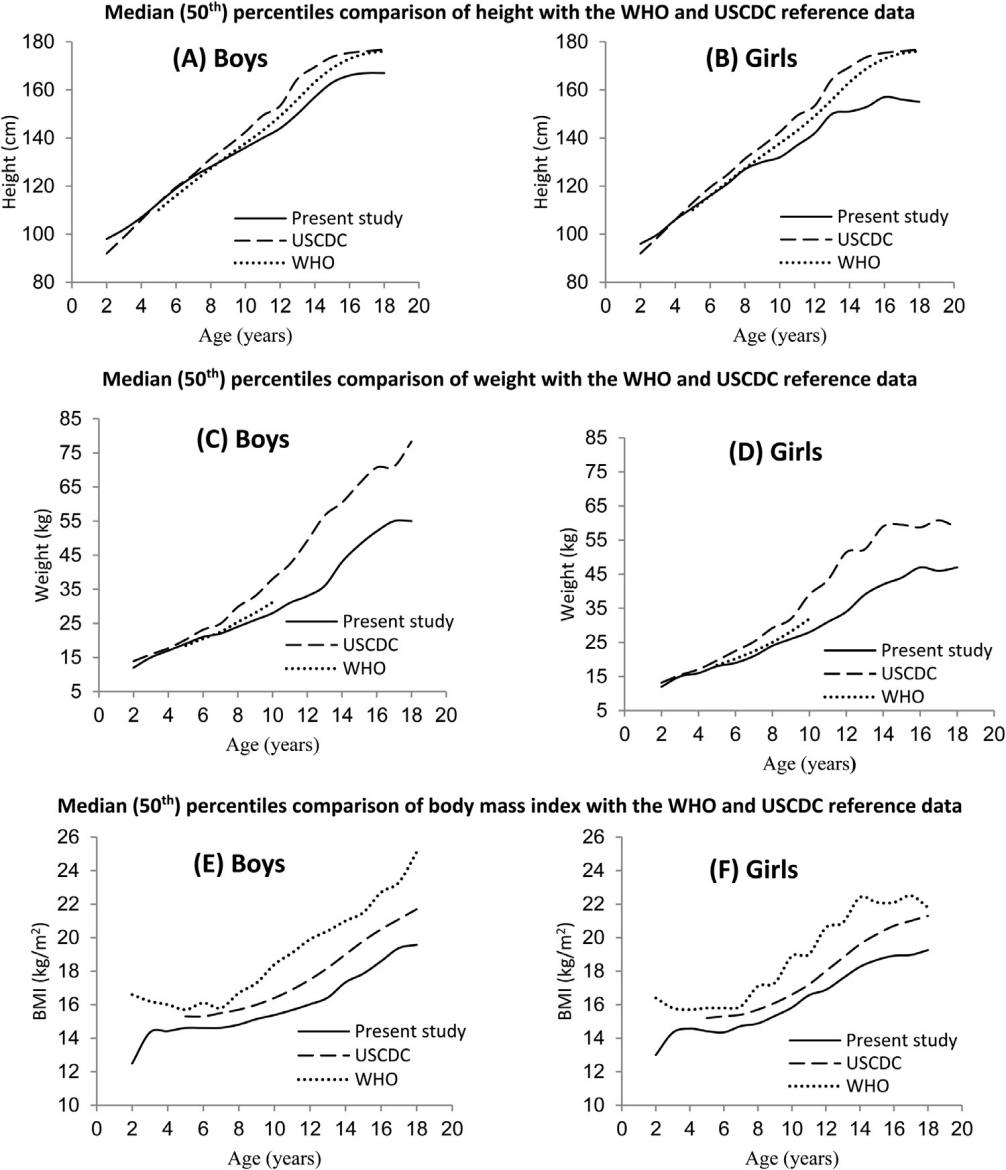
Comparison of 50th percentile curves of height (cm) (*A* + *B*), weight (kg) (*C* + *D*) and BMI (kg/m^2^) (*E* + *F*) for the Pakistani boys and girls with the WHO [5] and USCDC [6] reference data sets.

The socio-demographic characteristics-age, gender, residential city and all anthropometric characteristics were analyzed using descriptive statistics, i.e., frequencies along with their percentages and means ± standard deviation (SD). Socio-demographic and anthropometric data were analyzed using the “software”, Statistical Package for Social Sciences (SPSS) version 25.0. A total of 10,782 children and adolescents aged 2–19 years were enrolled in the dataset. In total, 5593 (51.9%) were boys and 5189 (48.1%) were girls. The collected raw data used for each table was stored in a Microsoft Excel Worksheet (.xlsx) format. The dataset was further divided into two age groups, i.e., (i) 2–10 years and (ii) 11–19 years age. Mostly participants 5840 (54.2%) belonged to the age-group of 11 to 19 years. The mean (± SD) age, height, weight, BMI, NC, WC, HpC, WHpR, WHtR, MUAC and WrC of the total participants were 10.63 (±4.03) years, 137.92 (±19.61) cm, 32.82 (±12.77) kg, 16.53 (±2.84) Kg/m^2^, 26.55 (±3.06) cm, 60.49 (±10.13) cm, 66.79 (±11.36) cm, 0.91 (±0.10) cm, 0.44 (±0.05) cm, 18.26 (±3.10) cm, and 13.20 (±1.79) cm, respectively (Table 1). The comparison of anthropometric characteristics by age and gender are shown in Table 2. For overall sample, results indicated that there was a significant (*p*< 0.01) difference in the mean values of height, weight, BMI, NC, WC, HpC, WHtR and WrC with respect to gender. While WHpR mean values were not statistically significant between boys and girls (i.e., boys vs*.* girls: 0.91±0.09 *vs.* 0.90±0.10; *p* = 0.376). We also compared the median (50th) percentile values with the WHO[5] and USCDC[6] growth references in Fig. 1. Except few early ages, the median percentiles of height and weight for the Pakistani boys and girls were lower than the WHO and USCDC reference values. While the BMI values in all ages were lower than the WHO and USCDC reference values. A significant disparity between our reported centiles and centile values of the WHO and USCDC show that each country needs to collect its own data of anthropometric parameters for studying the growth and nutritional status assessment in children and adolescents. Our anthropometric data of other parameters i.e., WC, WHpR, WHtR, MUAC, NC and WrC would also be helpful to local and regional pediatricians, nutritionists and other epidemiological researchers for other clinical findings.

**Table 1:** Socio-demographic characteristics and descriptive statistics of anthropometric parameters of all children and adolescents, aged 2–19 years.

Variables	N (%)
Total sample	10,782 (100)
*Gender status*	
Boys	5593 (51.9)
Girls	5189 (48.1)
Age (Years, mean ± *S*.D)	10.63 ± 4.03 (Range = 02–19)
*Age-groups (years)*	
2–10	4942 (45.8%)
11–19	5840 (54.2%)
*Residential city*	
Multan	2051 (19.0)
Lahore	5037 (46.7)
Rawalpindi or Islamabad	3694 (34.3)
Descriptive statistics	mean ± SD (Min-Max)
*Anthropometric parameters*	
Height (cm)	137.92 ± 19.61 (85.0–188.0)
Weight (kg)	32.82 ± 12.77 (10.0–74.0)
BMI (kg/m^2^)	16.53 ± 2.84 (8.66–24.52)
NC (cm)	26.55 ± 3.06 (16.51–36.83)
WC (cm)	60.49 ± 10.13 (35.56–91.44)
HpC (cm)	66.79 ± 11.36 (36.83–101.60)
WHpR (cm)	0.91 ± 0.10 (0.50–1.58)
WHtR (cm)	0.44 ± 0.05 (0.28–0.76)
MUAC (cm)	18.26 ± 3.10 (10.16–26.92)
WrC (cm)	13.20 ± 1.79 (8.64–18.80)

SD: Standard deviation; BMI: Body mass index; NC: Neck circumference; WC: Waist circumference; HpC: Hip circumference; WHpR: Waist-to-hip ratio; WHtR: Waist-to-height ratio; MUAC: Mid-upper-arm circumference; WrC: Wrist circumference.

**Table 2:** Comparison of anthropometric characteristics by age and gender.

		Gender				
Characteristics	Age-group (years)	N	Boys	N	Girls	p-value
*Height (cm)*	2–10	2078	123.23 ± 12.92	2864	120.15 ± 12.76	<0.0001
	11–19	3515	153.95 ± 12.64	2325	148.66 ± 10.36	<0.0001
	Total	5593	142.54 ± 19.56	5189	132.93 ± 18.41	<0.0001
*Weight (kg.)*	2–10	2078	23.40 ± 6.64	2864	22.24 ± 6.48	<0.0001
	11–19	3515	42.07 ± 10.97	2325	40.30 ± 8.99	<0.0001
	Total	5593	35.13 ± 13.17	5189	30.33 ± 11.83	<0.0001
*BMI (kg/m^2^)*	2–10	2078	15.15 ± 2.24	2864	15.12 ± 2.30	0.747
	11–19	3515	17.47 ± 2.73	2325	18.06 ± 2.72	<0.0001
	Total	5593	16.60 ± 2.79	5189	16.44 ± 2.89	0.003
*NC (cm)*	2–10	2078	24.63 ± 1.93	2864	24.27 ± 2.06	<0.0001
	11–19	3515	28.48 ± 2.71	2325	28.13 ± 2.41	<0.0001
	Total	5593	27.05 ± 3.08	5189	26.00 ± 2.94	<0.0001
*WC (cm)*	2–10	2078	54.57 ± 7.22	2864	53.52 ± 7.28	<0.0001
	11–19	3515	62.24 ± 10.32	2325	58.61 ± 9.58	<0.0001
	Total	5593	66.78 ± 9.13	5189	64.88 ± 8.28	<0.0001
*HpC (cm)*	2–10	2078	58.80 ± 7.38	2864	57.63 ± 7.08	<0.0001
	11–19	3515	74.37 ± 8.52	2325	73.73 ± 9.21	0.007
	Total	5593	68.58 ± 11.06	5189	64.85 ± 11.37	<0.0001
*WHpR*	2–10	2078	0.93 ± 0.10	2864	0.93 ± 0.09	0.810
	11–19	3515	0.90 ± 0.09	2325	0.88 ± 0.09	<0.0001
	Total	5593	0.91 ± 0.09	5189	0.90 ± 0.10	0.376
*WHtR*	2–10	2078	0.44 ± 0.05	2864	0.45 ± 0.06	0.061
	11–19	3515	0.43 ± 0.05	2325	0.44 ± 0.06	0.053
	Total	5593	0.43 ± 0.05	5189	0.44 ± 0.05	<0.0001
*WrC*	2–10	2078	12.20 ± 1.33	2864	11.90 ± 1.30	<0.0001
	11–19	3515	14.45 ± 1.55	2325	13.81 ± 1.37	<0.0001
	Total	5593	13.61 ± 1.83	5189	12.76 ± 1.63	<0.0001

BMI: Body mass index; NC: Neck circumference; WC: Waist circumference; HpC: Hip circumference; WHpR: Waist-to-hip ratio; WHtR: Waist-to-height ratio; WrC: Wrist circumference.

## 2 Experimental Design, Materials and Methods

A cross-sectional multi-ethnic anthropometric survey (MEAS) was carried out in the four major cities of Pakistan, i.e., Multan, Lahore, Rawalpindi and Islamabad (the capital city). In these cities, health care and educational facilities are very impressive, and job opportunities related to industrialization and public sector are also outstanding. Most of the dwellers in these are immigrants from the other regions of Pakistan. Thus, the population of the stated cities shows to be a good representation of the entire population of Pakistan [1]. The core purpose of this survey was to examine the nutritional status and somatic growth assessment of children and adolescents. The details of the sampled population and the sampling methodology of this survey has been described in a few previous articles [2,3]. Briefly, the survey included 10,782 children and adolescents aged 2 to 19 years and the dataset of 9929 children and adolescents aged 5–19 years were collected from different public and private schools (primary, secondary, and higher secondary). The complete list of schools (primary, secondary, and higher secondary) of the respective cities was taken from Punjab department of education (Schools). The random sampling was used for school selection. Further details about the data collection can be found in few previous studies [2,3].

## Ethics Statement

After elucidation, the objectives and nature of the investigation, a written consent from each school's head and a verbal consent were taken from each child's parents or guardians. They had the right to freely participate in or withdraw from the research project. The project was approved by the Departmental Ethics Committee of Bahauddin Zakariya University, Multan. Pakistan.

## Credit Author Statement

**Muhammad Asif:** Conceived idea, data collection, writing-original draft preparation, and analysis.

**Muhammad Aslam:** Supervision, **c**onceptualization, methodology, statistical analysis, and final review.

**Muhammad Qasim:** Writing- review & editing, original draft preparation and data collection.

**Saima Altaf:** Methodology, data curation, writing- original draft preparation.

**Amir Ismail:** Conceptualization, data curation, visualization.

**Hamza Ali**: Conceptualization, data curation.

## Declaration of Competing Interest

The authors declare that they have no known competing financial interests or personal relationships which have, or could be perceived to have, influenced the work reported in this article.

## Appendix Supplementary materials

Image, application 1
