# Supplementary material for: A dataset about anthropometric measurements of the Pakistani children and adolescents using a cross-sectional multi-ethnic anthropometric survey
Source: Data Brief. 2020 Dec 11;34:106642. doi: 10.1016/j.dib.2020.106642 (PMC7749368; doi:10.1016/j.dib.2020.106642)
Supplement: Supplementary file 1 [file mmc1.docx]

***Questionnaire***

**A Dataset about Anthropometric Measurements of the Pakistani Children and Adolescents Using a Cross-sectional Multi-ethnic Anthropometric Survey**

**Section-I**

**Socio-demographic information**

Age: __________ (years)

Gender status: (Boys/Girls)

Residential city: (Islamabad or Rawalpindi/ Lahore/ Multan)

**Section-II**

**Anthropometric measurements**

Height (cm): ___________Weight (kg): __________Waist circumference (cm): __________

Hip circumference (cm): _______________ Neck circumference (cm): _________________

Mid-upper arm circumference (cm): _________ Wrist circumference (cm): __________

Participant/Parent signature: __________________
